# hNET-targeted [^18^F]MFBG PET as baseline functional imaging in newly diagnosed neuroblastoma

**DOI:** 10.7150/thno.139324

**Published:** 2026-08-24

**Authors:** Lifeng Zhang, Xinhui Su, Xinyue Zheng, En Ren, Li Hou, Junqing Mao, Tingting Zhang, Jinhu Wang, Jingjing Zhang, Peipei Wang

**Affiliations:** 1 Department of Surgical Oncology, Children's Hospital, Zhejiang University School of Medicine, National Clinical Research Center for Child Health, Hangzhou, China.; 2 Department of Nuclear Medicine, The First Affiliated Hospital, Zhejiang University School of Medicine, Hangzhou, China.; 3 Department of Diagnostic Radiology, Yong Loo Lin School of Medicine, National University of Singapore, Singapore, Singapore.; 4 Theranostics Centre of Excellence, Yong Loo Lin School of Medicine, National University of Singapore, Singapore, Singapore.; 5 Clinical Imaging Research Centre, Centre for Translational Medicine, Yong Loo Lin School of Medicine, National University of Singapore, Singapore, Singapore.

**Keywords:** [^18^F]MFBG, PET, neuroblastoma, human norepinephrine transporter, initial stage

## Abstract

**Rationale:**

[^18^F]meta-fluorobenzylguanidine ([^18^F]MFBG) is an human norepinephrine transporter (hNET)-targeted PET tracer that has shown superior lesion detection than [^123^I]MIBG SPECT in patients with relapsed or refractory neuroblastoma. Its value in newly diagnosed patients, however, has not been well established. We therefore performed this study to evaluate the additional staging information of [^18^F]MFBG beyond comprehensive anatomic imaging and assessed its agreement with [^18^F]FDG PET at baseline.

**Methods:**

We prospectively enrolled consecutive children with newly diagnosed neuroblastoma. All patients underwent baseline [^18^F]MFBG PET and comprehensive anatomic imaging, including contrast-enhanced CT and/ or MRI from the neck to the pelvis and whole-body ultrasonography. The imaging studies were interpreted independently, and lesion distribution and International Neuroblastoma Risk Classification (INRG) stage were recorded. A subgroup also underwent paired [^18^F]FDG PET for functional comparison. Paired detection rates were compared using the McNemar test. Intermodality concordance was evaluated using Cohen κ statistics, and Curie scores were compared using Wilcoxon signed-rank tests.

**Results:**

Forty patients (median age, 23.5 months) were analyzed. The primary tumors were mainly located in the abdomen or pelvis (36/40, 90%), with corresponding imaging findings including contrast-enhanced abdominopelvic CT and MRI, non-contrast cervicothoracic CT, and whole-body ultrasonography. [^18^F]MFBG PET identified bone marrow metastases more frequently than anatomic imaging (65% vs. 32.5%, P = 0.001; κ = 0.41), leading to stage reclassification in 20% of cases and treatment modification in 10% based on tumor-board review. Even within overlapping fields of view (neck to proximal femur), [^18^F]MFBG maintained superior bone marrow detection rates (55% vs. 32.5%, P = 0.004; κ = 0.56). Among the patients initially classified as L2, half were upstaged based on hNET-targeted PET findings. In the paired [^18^F]FDG PET subset (n = 26), the patient-level INRG stage was fully concordant between modalities. However, lesion-level analysis revealed a higher bone marrow disease burden on [^18^F]MFBG (191 vs. 140, P = 0.002), while FDG detected 13% additional nodal lesions in five patients.

**Conclusion:**

In newly diagnosed neuroblastoma, hNET-targeted [^18^F]MFBG PET detected clinically relevant bone marrow involvement not identified by comprehensive anatomic imaging, resulting in stage reclassification and treatment modification in a subset of patients. Paired [^18^F]FDG PET demonstrated patient-level stage concordance with lesion-level heterogeneity.

## Introduction

Neuroblastoma is the most common extracranial solid tumor in children, representing approximately 15% of pediatric cancer-related deaths 1-3. Precise initial staging is crucial for risk stratification and treatment planning, given the significant therapeutic variations between localized, intermediate-risk, and high-risk disease categories 4-6.

Current staging approaches rely on a multimodal workup that combines anatomic imaging (CT, MRI, and whole-body ultrasonography), bilateral bone marrow assessment, and functional imaging. Human norepinephrine transporter (hNET)-targeted [^123^I]meta-iodobenzylguanidine ([^123^I]MIBG) scintigraphy remains the standard functional imaging for neuroblastoma 7-12. However, tracer availability and examination logistics may limit its routine use 13-16. [^18^F]FDG PET has also been increasingly used for baseline evaluations because of its wilder availability and reported sensitivity in newly diagnosed patients 13.

[^18^F]Meta-fluorobenzylguanidine ([^18^F]MFBG) is a PET-based tracer targeting hNET that allows for whole-body imaging in a single PET/CT or PET/MRI scan, offering high spatial resolution and quantitative capabilities. Recent findings have shown that [^18^F]MFBG PET can detect all lesions identified by [^123^I]MIBG and reveal additional lesions in patients with neuroblastoma, mainly with relapsed or refractory patients. These studies primarily focused on comparing lesion detection and diagnostic performance 17-21. Dosimetry analyses have also confirmed the feasibility of using [^18^F]MFBG. The estimated effective dose of [^18^F]MFBG is 0.019–0.023 mSv/MBq, comparable to that of [^123^I]MIBG (~0.017–0.019 mSv/MBq) 20, 22, and substantially lower than that of [^124^I]MIBG PET (~0.58–1.03 mSv/MBq) 23. Consequently, the role of [^18^F]MFBG PET in the initial staging of newly diagnosed patients with neuroblastoma remains uncertain. In particular, the added value of [^18^F]MFBG PET beyond comprehensive anatomic imaging for evaluating metastatic burden remains unclear, as does the need for additional [^18^F]FDG PET to detect hNET-non-avid disease at diagnosis. Therefore, further research is required to determine the added value of hNET-targeted PET imaging in the initial staging process.

We investigated whether [^18^F]MFBG PET is sufficient to serve as a feasible baseline functional imaging strategy for newly diagnosed neuroblastoma. Specifically, we assessed its added staging value beyond comprehensive anatomic imaging, its impact on initial treatment planning, and, in a paired subset, whether [^18^F]FDG PET identified clinically meaningful discordant disease that could alter staging or risk assignment.

## Methods

### Study Design and Population

This single-center prospective study aimed to evaluate the utility of [^18^F]MFBG PET in the initial staging of newly diagnosed neuroblastomas. Patients were consecutively enrolled between August 2024 and January 2026. Inclusion criteria comprised histologically confirmed or clinically highly suspected neuroblastoma, absence of prior systemic antitumor therapy before imaging, and completion of baseline comprehensive anatomic imaging within two weeks before PET. Patients with inadequate PET image quality were excluded. The subset undergoing paired [^18^F]FDG PET was selected based on logistical availability, and agreed upon by patients and guardians within one week of [^18^F]MFBG PET. The primary endpoint was the proportion of patients with stage reclassification using [^18^F]MFBG PET compared to comprehensive anatomic imaging. Key secondary endpoints included differences in bone marrow metastasis detection rate, concordance with [^18^F]FDG, and the proportion of treatment modifications attributed to PET findings. The study received approval from the Institutional Review Board of our hospital (2025 C-IIT No.008) and was registered at ClinicalTrials.gov (NCT06852807). Written informed consent was obtained from the legal guardians of all patients and from patients aged 8 years and older.

### Comprehensive Anatomic Imaging Protocols

All patients underwent comprehensive anatomic imaging as part of the initial staging. This included contrast-enhanced CT and MRI with/without contrast of the primary site and whole-body ultrasonography of the neck, chest, abdomen, and pelvis, covering the neck to the pelvis. Imaging protocols followed institutional standards and national pediatric oncology guidelines 9. Specifically, patients with mediastinal primaries underwent cervicothoracic contrast-enhanced CT angiography (CTA), whereas those with retroperitoneal or pelvic primaries underwent abdominopelvic CTA and enhanced MRI combined with cervicothoracic non-contrast chest CT. Cranial MRI was performed in patients with suspected intracranial extension.

### [^18^F]MFBG and [^18^F]FDG PET Protocol

[^18^F]MFBG was synthesized as previously described 24, 25, and [^18^F]FDG was synthesized using an automatic synthesis module (TracerLab Fx FDG; GE Healthcare) at our hospital. The tracer was administered intravenously at 2–3 MBq/kg. Whole-body PET acquisition was performed 60–90 min after injection, covering the vertex to the toes, using either a dedicated PET/CT scanner (Biograph Vision 600, Siemens, Germany) or PET/MR (Signa, GE Healthcare, USA). For PET/CT, a single low-dose CT scan (120 kVp, 10–30 mA) was performed to correct for attenuation. For the PET/MRI, a two-point Dixon sequence was used for attenuation. PET scans were acquired in the three-dimensional mode with 3 min per bed position for the torso and 2 min for the lower limbs. Images were reconstructed using a vendor-provided ordered-subset expectation maximization algorithm (3 iterations, 10 subsets) with corrections for attenuation, scatter, and randomness, similar to the clinical [^18^F]FDG protocols.

### Image Interpretation

Apart from basic clinical information (patient age, sex, and indication for imaging), PET and anatomic imaging were independently interpreted by separate readers who were blinded to the findings of the alternative imaging modality. CT and MRI scans were reviewed by two pediatric radiologists (Y.C. and F.D., with 15 and 18 years of experience), and ultrasonography by one expert pediatric radiologist (B.X., 25 years). The [^18^F]MFBG PET studies were interpreted by three nuclear medicine physicians (P.W. T.Z. and X.S., with 10, 15 and 25 years of experience). A lesion was considered positive if the focal uptake exceeded the background levels in a nonphysiological distribution consistent with metastatic spread. Discrepancies among readers were resolved by a majority vote (at least two of three readers). No indeterminate or missing imaging findings were observed. PET-positive lesions without corresponding cortical destruction on CT/MRI are described as bone marrow involvement, whereas lesions demonstrating structural bone changes are referred to as osseous metastases. The findings were subsequently discussed by a tumor board specializing in neuroblastoma. For each patient, readers recorded the presence and distribution of (1) the primary tumor, (2) regional lymph nodes, (3) distant lymph nodes (cervical, thoracic, abdominal, and pelvic), with lesion matching based on the corresponding CT/MR images. Clustered metastatic lymph nodes within the same anatomical region were counted as up to three lesions. (4) bone marrow metastases (skull, spine, pelvis, thoracic cage, and extremities), and (5) other soft tissue lesions (e.g., liver, lung, kidney, and skin). Given the presence of disseminated osseous and bone marrow metastases in neuroblastoma, regional semi-quantification was performed using Curie score segments for [^18^F]MFBG and [^18^F]FDG PET 26. Although the Curie scoring system was originally developed and validated for [^123^I]MIBG imaging, region-based bone marrow scoring has been adopted in prior PET-based neuroblastoma studies to quantify diffuse marrow involvement, which is often not reliably countable as discrete lesions 27, 28.

### Reference Standard

Histopathological confirmation was obtained from the biopsy specimens of primary or metastatic lesions in all patients. Bone marrow aspiration/biopsy was performed in all patients at diagnosis, usually at the time of tumor biopsy or surgical resection, with bilateral iliac crest sampling regardless of imaging findings. Hematoxylin-eosin and immunohistochemical staining for GD2 were conducted to detect marrow involvement. Genetic analyses for MYCN amplification and 11q deletion were carried out following institutional protocols.

A comprehensive reference standard was utilized to determine metastatic disease and establish final staging through discussions in a multidisciplinary tumor board and subsequent imaging follow-up. This standard encompasses (1) histopathology or bone marrow biopsy results, (2) all anatomic imaging and PET findings, (3) follow-up imaging, and (4) treatment response during clinical follow-up. Imaging findings were deemed true positives when corroborated by pathology, clear progression on follow-up imaging, or a consistent response post systemic therapy. The ultimate INRG stage and risk group classification were assigned by integrating imaging findings, pathology, bone marrow biopsy, and follow-up data, as determined by a multidisciplinary pediatric oncology tumor board.

### Statistical Analysis

No formal a priori sample size calculation was performed. This study was designed as a prospective exploratory cohort including all consecutive eligible patients during the predefined study period to evaluate the clinical workflow impact of [^18^F]MFBG PET at baseline. The paired [^18^F]FDG PET subset was determined by clinical availability, physician discretion, and patient willingness as part of baseline evaluation. Continuous variables were presented as mean ± standard deviation or median (interquartile range), while categorical variables were presented as counts and percentages. The McNemar test was utilized for paired comparisons of lesion detection rates between comprehensive anatomic imaging and [^18^F]MFBG PET, as well as between [^18^F]MFBG and [^18^F]FDG PET in the subset cohort. The Wilcoxon signed-rank test was employed to evaluate differences in the Curie scores between the PET modalities. Analyses of the regional Curie scores and nodal compartments were exploratory, with no multiple comparison adjustments applied. Cohen’s κ statistic was calculated to assess inter-modality agreement. Agreement was interpreted according to standard criteria (κ < 0.20, poor; 0.21–0.40, fair; 0.41–0.60, moderate; 0.61–0.80, substantial; >0.80, almost perfect). All statistical tests were two-sided, and a significance level of P < 0.05 was considered statistically significant. Statistical analyses were conducted using SPSS Statistics (version 32.0; IBM, Armonk, NY, USA) and GraphPad Prism (version 9.0; GraphPad Software, San Diego, CA, USA).

## Results

### Clinical Characteristics of Patients

**Figure 1:**
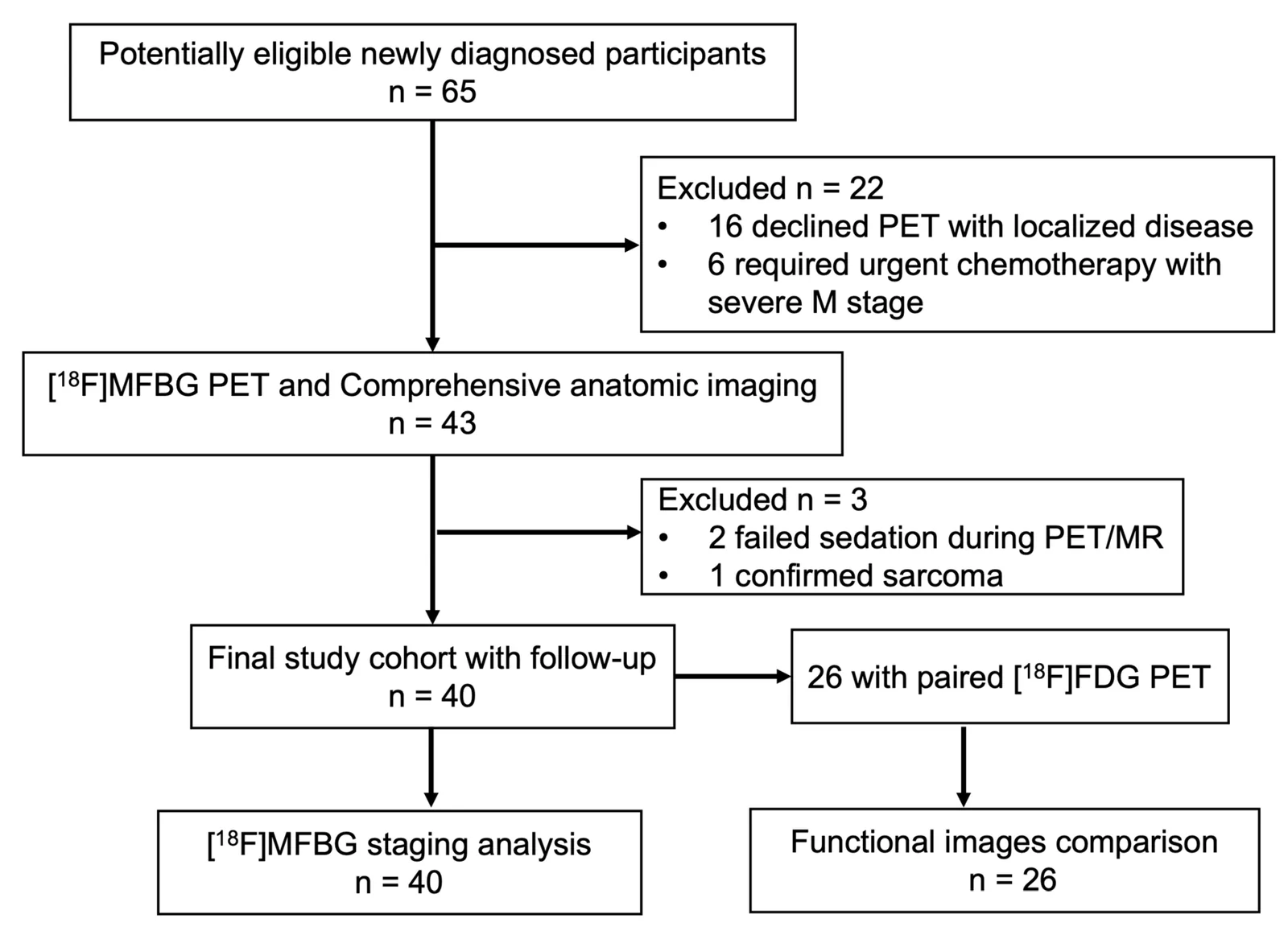
Flow diagram showing details of the patient selection process. [^18^F]MFBG: [^18^F]meta-fluorobenzylguanidine; [^18^F]FDG: [^18^F]fluorodeoxyglucose; PET: positron emission tomography.

Forty patients (median age: 23.5 [IQR: 8-43] months; 18 boys [45%] and 22 girls [55%]) were included in the final study cohort **(Figure 1)**. All patients had pathologically confirmed neuroblastoma, with 35 (87.5%) having neuroblastoma and 5 (12.5%) having ganglioneuroblastoma. The median follow-up duration was 8 months (range: 3–15 months). Patient characteristics are summarized in **Table 1**.

The primary tumors were most commonly located in the retroperitoneum (35/40, 87.5%). Thirty-six patients (90.0%) with abdominal or pelvic primaries underwent abdominopelvic contrast-enhanced CT angiography and MRI combined with non-contrast cervicothoracic chest CT and whole-body ultrasonography. Three patients with mediastinal primaries underwent cervicothoracic contrast-enhanced CTA and MRI combined with abdominal CT or MRI and whole-body ultrasonography. Six patients underwent cranial MRI based on the clinical indications. Thus, comprehensive anatomic imaging covered the torso from the neck to the proximal femur in all patients. In one patient, no primary lesion was identified on either [^18^F]MFBG or [^18^F]FDG PET.

Bone marrow biopsy results were positive in 11 patients (27.5%). MYCN amplification was present in seven (17.5%) patients, and 11q deletion in nine (22.5%) patients. The final INRG stage distribution based on the composite reference standard was L1 in two patients (5.0%), L2 in seven (17.5%), MS in five (12.5%), and M in 26 patients (65.0%). All patients tolerated the PET imaging without adverse events or sedation-related complications.

### Patient-Level Comparison of [^18^F]MFBG and Anatomic Imaging

In one patient (2.5%), the primary site was not identifiable in all images. Primary tumors were detected in 39 patients using comprehensive anatomic imaging. [^18^F]MFBG PET identified the primary lesion in all 36 patients who did not undergo surgical resection before PET. Regional lymph node metastases were observed in 29 of the 40 patients (72.5%) on comprehensive anatomic imaging and PET. Distant lymph node metastases were detected in 18 patients, including 17 identified using anatomic imaging and 15 using PET. At the patient level for distant nodal disease, 14 patients were positive and 22 were negative for both modalities. Discordant findings were observed in four patients: three patients were positive on anatomic imaging but negative on MFBG PET, and one patient was negative on anatomic imaging but positive on MFBG PET.

**Figure 2:**
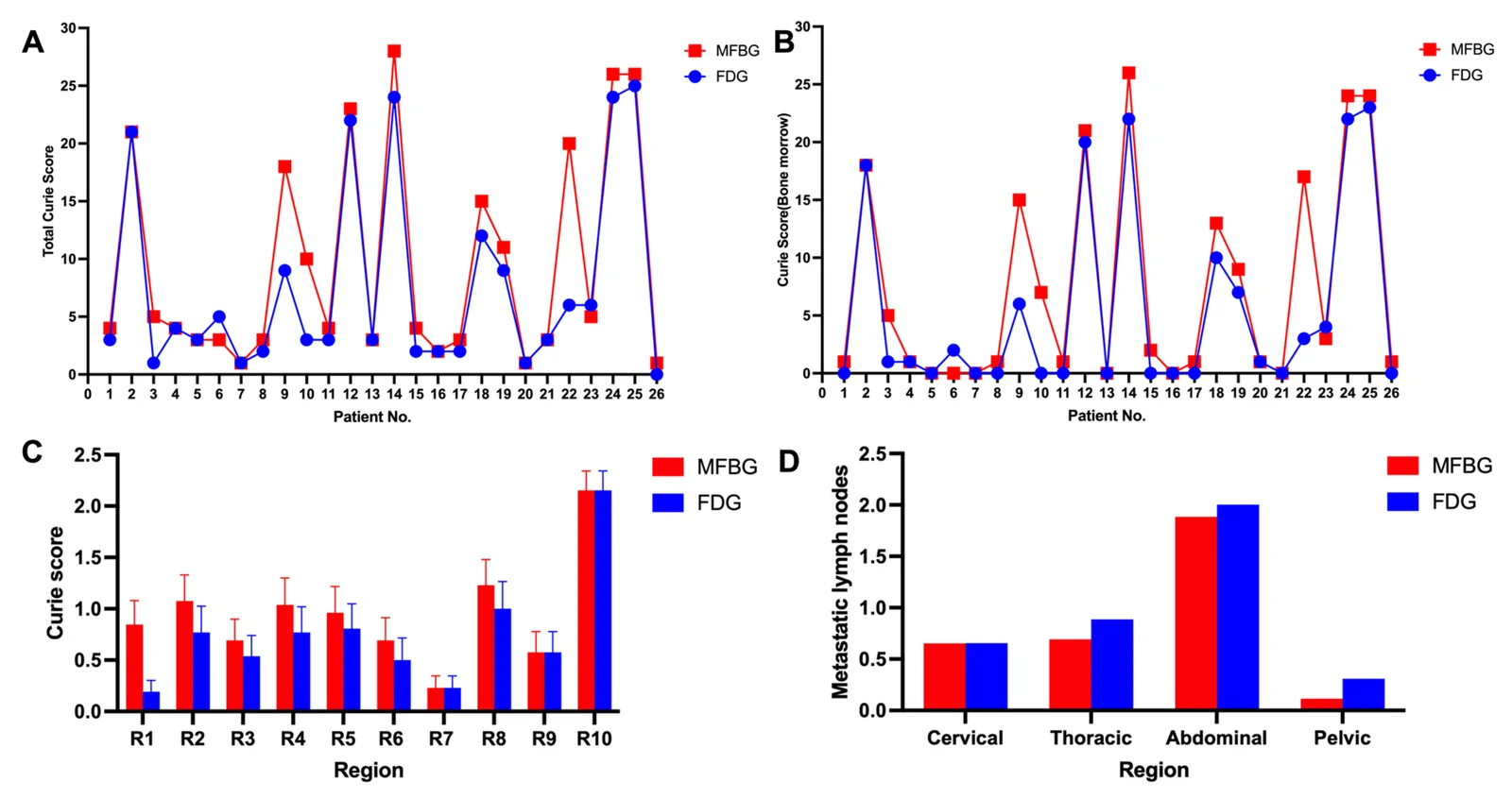
** [^18^F]MFBG and [^18^F]FDG PET/CT comparison.** (A) Total Curie scores for each of the 26 patients, comparing [^18^F]MFBG (red squares) and [^18^F]FDG (blue circles). (B) Bone marrow disease burden (R1–R9) Curie scores for each patient. (C) Mean regional Curie scores for R1–R10 (R1–R9: bone marrow regions; R10: soft tissue) in the study cohort, shown as mean ± SEM. (D) Number of metastatic lymph nodes detected by [^18^F]MFBG and [^18^F]FDG in cervical, thoracic, abdominal, and pelvic regions.

**Figure 3:**
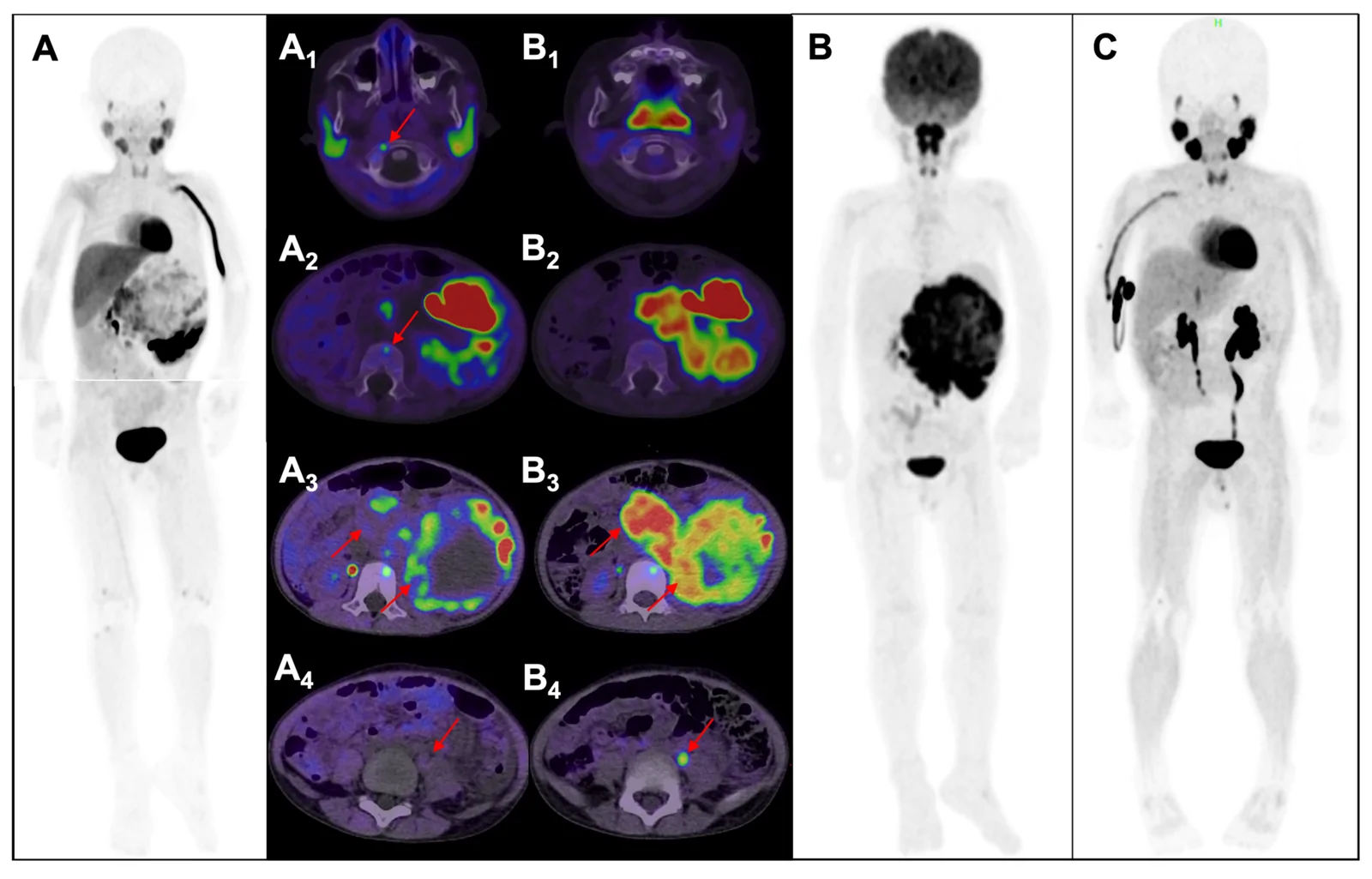
** [^18^F]MFBG PET showing bone marrow involvement not detected by [^18^F]FDG PET in a pediatric patient with newly diagnosed neuroblastoma.** A 4-year-old boy presenting with fever and abdominal pain. Anatomic imaging (abdominal CTA and abdominopelvic MRI) revealed a large retroperitoneal mass suggestive of neuroblastoma. [^18^F]MFBG PET/CT MIP (A) and axial PET/CT fusion images demonstrated MFBG-avid lesions in the C1 vertebra (A₁) and L2 vertebra (A₂), as well as heterogeneous MFBG uptake within the primary tumor (A₃). However, an enlarged pelvic lymph node shows no appreciable MFBG uptake, consistent with a false-negative lesion (A₄). [^18^F]FDG PET/CT MIP (B) and corresponding axial fusion images show the absence of FDG uptake in C1 (B₁) and L2 (B₂), positive FDG uptake in the primary tumor and lymph node lesions (B₃ and B_4_). Follow-up [^18^F]MFBG PET/MR (C) after therapy shows no residual abnormal uptake.

Bone marrow involvement was observed in 26 of 40 patients (65.0%) on [^18^F]MFBG PET, compared with 13 of 40 (32.5%) on comprehensive anatomic imaging (P = 0.001; κ = 0.41). All anatomically positive bone marrow lesions were visualized using PET. Within overlapping fields of view, detection rates remained significantly higher for MFBG (55% vs. 32.5%, P = 0.004; κ = 0.56). Despite moderate agreement, patient-level discrepancies were observed, primarily reflecting additional skeletal marrow lesions detected by [^18^F]MFBG PET. Anatomic imaging primarily detected lesions in the thoracolumbar spine and pelvis. PET revealed additional lesions in the skull (12/40, 30.0%) and extremities (9/40, 22.5%), which were outside the routine anatomic imaging field of view **(Figure 2 and 3)**. Bilateral bone marrow aspiration/biopsy confirmed bone marrow involvement in 11 of the 40 patients (27.5%) in the PET-positive group. Notably, the comprehensive anatomic imaging protocols used in this study did not routinely include the skull or distal extremities, whereas [^18^F]MFBG PET provided vertex-to-toe coverage. Regionally, MFBG PET detected more bone marrow lesions across all evaluated compartments **(Table 2)**.

Other metastatic sites were identified in 6 of the 40 patients (15.0%) using both modalities. Specifically, liver metastases were detected in 4 patients (10.0%) using both modalities. Anatomic imaging revealed multiple subcentimeter pulmonary nodules in one patient (2.5%). These lesions showed no tracer uptake on MFBG PET but resolved completely following induction chemotherapy, confirming pulmonary metastases. This patient did not undergo paired FDG PET. Renal and pleural involvement were identified in one patient (2.5%) using both modalities*.*

### Paired Functional Imaging Comparison: [^18^F]MFBG versus [^18^F]FDG

In the paired functional subset of 26 patients, the patient-level INRG stage and risk assignments were completely consistent between [^18^F]MFBG and [^18^F]FDG PET (L1, n = 1; L2, n = 3; M, n = 19; MS, n = 3), showing no discrepancies at baseline. On a global disease level, MFBG revealed higher total Curie scores compared to FDG (median, 4 [IQR, 3-18.5] vs. 3 [IQR, 2-9.75]; P < 0.001) **(Figure 2A)**.

**Figure 4:**
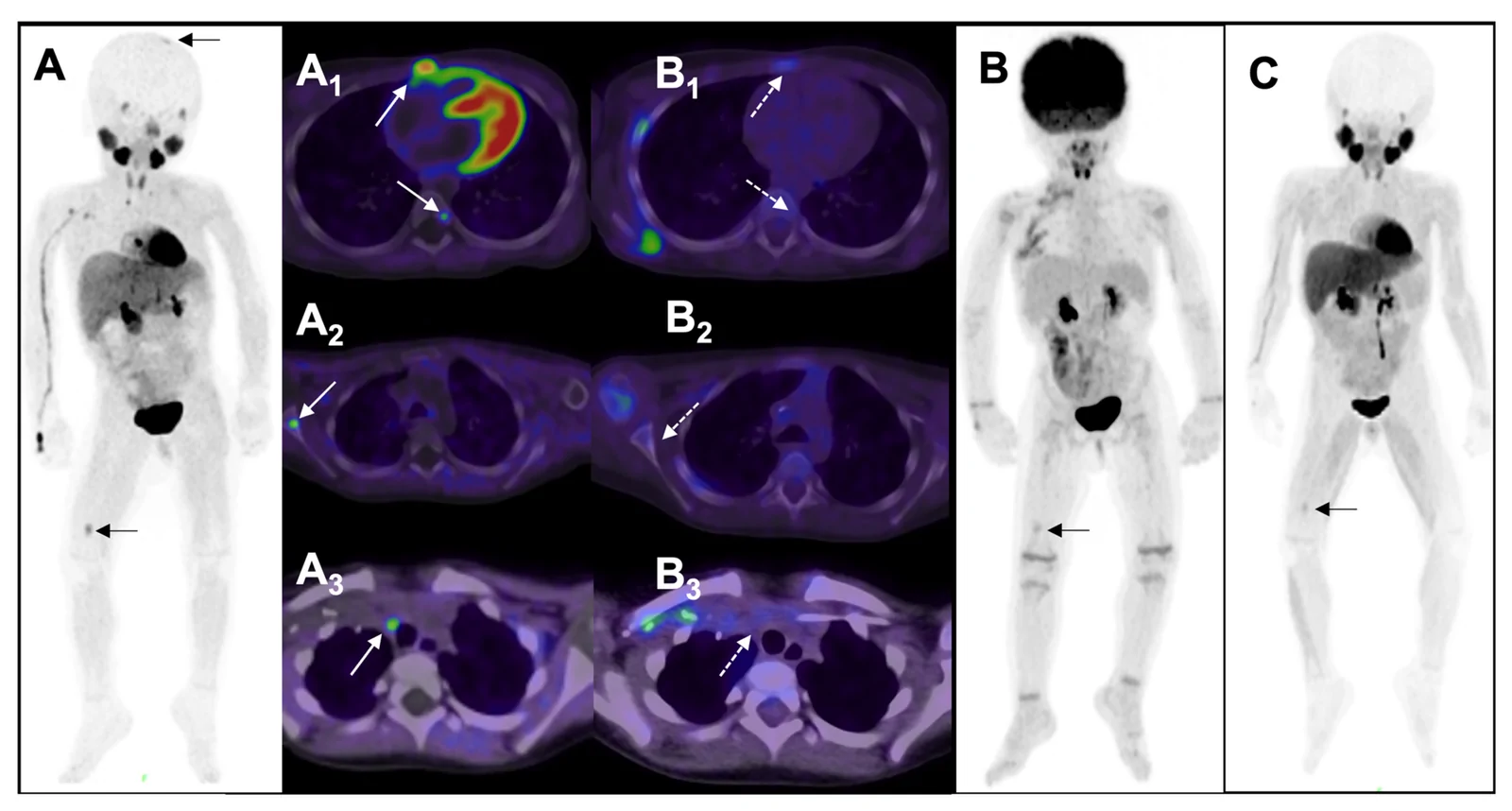
** [^18^F]MFBG PET showing occult bone marrow metastases not detected through comprehensive anatomic imaging or [^18^F]FDG PET in newly diagnosed neuroblastoma.** A 2-year-old girl with pathologically confirmed differentiating neuroblastoma resected from the mediastinum. Comprehensive anatomic imaging revealed no evidence of metastatic disease. Bone marrow biopsy and genetic testing (including MYCN and 11q status) were negative. [^18^F]MFBG PET/CT MIP (A) demonstrates multiple foci of abnormal uptake (arrows). Axial PET/CT fusion images show additional MFBG-avid lesions in the sternum and T7 vertebra (A₁), right scapula (A₂), and anterior chest wall adjacent to the prior surgical site (A₃), none of which demonstrate corresponding structural abnormalities on CT. Corresponding [^18^F]FDG PET/CT MIP (B) and fusion images (B₁–B₃) show no abnormal uptake at these sites. Based on [^18^F]MFBG PET findings, INRG stage was revised from L2 to M, and risk classification escalated from intermediate to high risk. (C) Follow-up [^18^F]MFBG PET MIP after high-risk induction therapy demonstrates partial response.

Bone involvement was detected in 20 out of 26 patients (76.9%) using MFBG, in comparison to 14 out of 26 patients (53.8%) using FDG (P = 0.07; κ = 0.36). Concordance analysis indicated that 13 patients tested positive for both imaging modalities, 7 were positive only for MFBG, 1 was positive only for FDG, and 5 were negative for both. MFBG also revealed a higher bone marrow disease burden than FDG (median Curie score, 1.5 [IQR, 0.75-15.5] vs. 1 [IQR, 0-7.75]; total bone marrow Curie score, 191 vs. 140, P = 0.002) **(Figure 2B)**. Regional analysis showed a notably higher Curie score in R1 (craniofacial) with MFBG compared to FDG (22 vs. 5; P = 0.007). No statistically significant differences were observed in the other regions **(Figure 2C)**. Representative examples of discordant bone marrow findings are illustrated in **Figures 3 and 4**.

Across the paired functional and anatomic imaging, 100 metastatic lymph node lesions were identified in the subset of 26 patients. The regional distribution did not significantly differ between the modalities: cervical (17 vs. 17), thoracic (18 vs. 23), abdominal (49 vs. 52), and pelvic (3 vs. 8) (all P > 0.05) **(Figure 2D)**. Although FDG detected numerically more nodal lesions overall (13 additional lesions, 13%) in five patients, particularly within the calcified mediastinal and pelvic nodes, these discrepancies did not result in differences in patient-level INRG stage or risk assignment. The detection of other soft tissue metastases was identical, including two hepatic metastases and one pleural metastasis.

**Figure 5:**
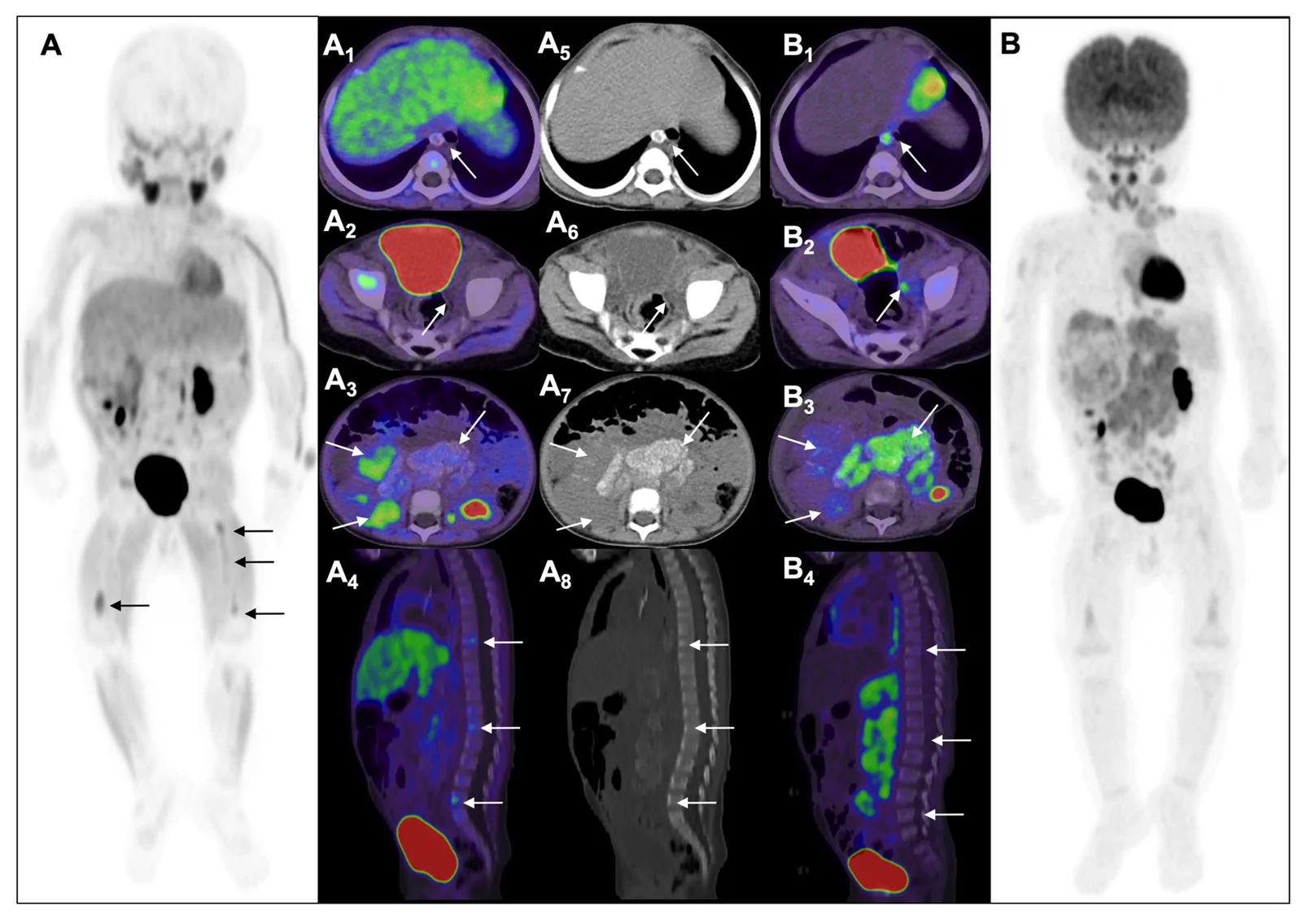
** Divergent functional phenotypes between NET-targeted [^18^F]MFBG PET and [^18^F]FDG PET in an 11-month-old boy with newly diagnosed neuroblastoma.** [^18^F]MFBG PET image (A) demonstrating diffuse bone marrow involvement in both lower extremities (arrows). Axial PET/CT fusion images show no MFBG uptake in enlarged calcified mediastinal (A_1_) and pelvic lymph nodes (A_2_). The retroperitoneal primary tumor (A_3_) demonstrates heterogeneous MFBG uptake, with relatively lower uptake in calcified components and higher uptake in soft-tissue portions. Sagittal fusion image (A_4_) demonstrates multiple focal MFBG-avid lesions. Corresponding CT images (A_5_–A_8_) show calcified lymph nodes and primary tumor without osseous destruction. In contrast, [^18^F]FDG PET MIP (B) and fusion images demonstrate intense uptake in lymph nodes (B_1_, B_2_), heterogeneous uptake in primary tumor (B_3_), however, no focal uptake on spine (B_4_). The lower extremities’ uptake appeared to be symmetric, making the distinction between marrow metastases and reactive changes uncertain. Following four cycles of induction chemotherapy, the patient underwent radical tumor resection with systematic lymph node dissection. Pathology confirmed metastatic neuroblastoma in both the calcified mediastinal and pelvic lymph nodes.

Overall, these findings illustrate distinct functional phenotypes, with MFBG emphasizing bone marrow disease and FDG exhibiting relatively higher avidity in nodal disease, as depicted in **Figure 5**.

### Staging and Clinical Management Changes

**Figure 6:**
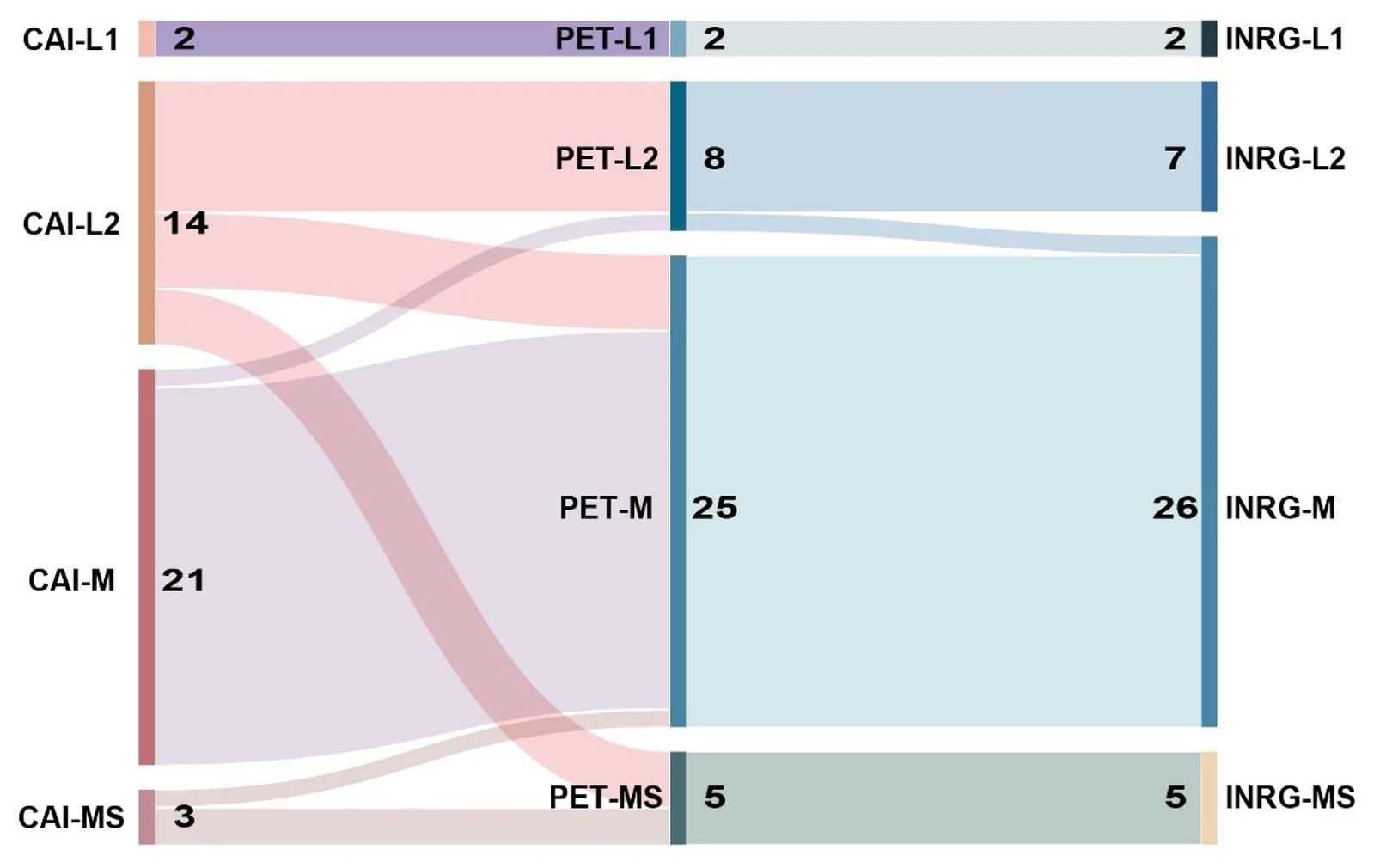
Three-tier Sankey diagram showing stage transitions from comprehensive anatomic imaging (CAI) to [^18^F]MFBG PET and final clinical staging in 40 patients with newly diagnosed neuroblastoma. Each flow line represents one patient. The left column shows INRG stage assignments based on CAI, the middle column shows stage reclassification after incorporation of [^18^F]MFBG PET findings, the right column shows the final stage determined by the composite reference standard, integrating PET, anatomic imaging, histopathology, and clinical/genetic data.

Using the INRG stage assigned from comprehensive anatomic imaging as the baseline, we reassessed stage after review of [^18^F]MFBG PET. Seven of the 14 patients (50%) initially classified as L2 were reclassified, including four to M and three to MS **(Figure 4)**. One of the three patients (33%) initially classified as MS was reclassified to M because [^18^F]MFBG PET showed extensive bone marrow involvement. Among the 21 patients classified as M on anatomic imaging, 20 remained M, whereas one was reclassified to L2 because the calcified mediastinal lymph nodes identified on CT showed no abnormal [^18^F]MFBG uptake. Overall, eight patients (20%) were upstage, mainly because of additional bone marrow disease detected on PET. In all eight cases, the final multidisciplinary stage was consistent with the PET-based classification. Stage transitions are shown in **Figure 6**.

Treatment modification occurred in four patients (10%). Three of the four patients were reclassified from L2 to M, and the single patient reclassified from MS to M underwent escalation to high-risk therapy. The patient was reclassified from M to L2, remained in the intermediate-risk category, and treatment was unchanged.

## Discussion

In this prospective cohort of children with newly diagnosed neuroblastoma, [^18^F]MFBG PET provided additional information beyond comprehensive anatomic imaging, mainly by the detection of bone marrow involvement, resulting in stage reclassification and treatment modification in a subset of patients. In a subgroup, [^18^F]MFBG and [^18^F]FDG PET yielded the same patient-level INRG stage, although the distribution of lesions differed between the two tracers. These findings support the utility of [^18^F]MFBG PET as an effective functional imaging at baseline in neuroblastoma.

The present study was not designed to establish equivalence, superiority, or replacement of guideline-recommended [^123^I]MIBG scintigraphy/SPECT. Rather, it evaluated the clinical role of next-generation hNET-targeted PET during baseline staging of newly diagnosed neuroblastoma. From a clinical workflow perspective, PET-based imaging facilitates more streamlined baseline evaluation, which may be particularly practicable in newly diagnosed children requiring prompt treatment initiation. [^123^I]MIBG remains the guideline-recommended functional imaging modality, supported by extensive clinical experience and its established role in selecting patients for [^131^I]MIBG radionuclide therapy.

The main additional finding provided by [^18^F]MFBG PET is more extensive detection of bone marrow involvement, directly impacting INRG stage and risk stratification. This difference remained significant even when the analysis was restricted to the overlapping imaging fields from the neck to the proximal femur (55% vs. 32.5%, P = 0.004; κ = 0.56), suggesting that the benefit was not solely due to extended coverage but also to the inherent sensitivity for bone marrow disease. Bone marrow aspiration and biopsy are standard procedures for initial evaluation, but iliac crest sampling may miss patchy or multifocal disease 29, 30. In our cohort, the identification of additional bone marrow involvement led to stage reclassification in 20% of patients and treatment modification in 10%. Notably, half of the initial L2 patients (7/14) were reclassified after review of the PET findings. These results emphasize the importance of accurately characterizing marrow disease as a crucial factor in initial risk assessment.

For soft-tissue metastases, [^18^F]MFBG and comprehensive anatomic imaging produced largely similar findings. Hepatic, renal, and pleural metastases demonstrated intense [^18^F]MFBG uptake. By contrast, multiple small pulmonary nodules in one patient showed no abnormal uptake, yet their complete resolution after chemotherapy confirmed metastases. This case highlights a potential limitation of hNET-targeted imaging for small lung lesions and underscores the importance of careful correlation with the chest CT when interpreting [^18^F]MFBG PET/CT at baseline. No definite false-positive [^18^F]MFBG findings were identified using the composite reference standard.

The paired functional PET analysis showed complete agreement in patient-level INRG stage, despite differences in lesion distribution. [^18^F]MFBG demonstrated a higher extent of bone marrow disease burden, particularly in the craniofacial region, where elevated physiological cerebral glucose uptake may reduce the visibility of adjacent skull lesions on [^18^F]FDG-PET. Conversely, [^18^F]FDG identified 13% more nodal lesions, mainly in calcified mediastinal and pelvic lymph nodes. The [^18^F]FDG-positive/[^18^F]MFBG-negative lymph nodes were surgically resected and confirmed as metastatic neuroblastoma. However, these nodes were also visible on anatomic imaging and did not change patient-level stage. Our findings therefore do not indicate the preferential use of [^18^F]FDG for baseline soft-tissue assessment. Instead, they suggest that [^18^F]FDG may provide complementary metabolic information in selected cases. Previous studies has reported higher lesion detection with [^18^F]MFBG than with [^18^F]FDG, particularly in patients after treatment 31. This current study is distinct in its exclusive focus on newly diagnosed patients, where disease distribution and tumor characteristics may differ from those in treated populations. Although the two PET tracers showed lesion-level discordance, no patient-level stage discordance was observed. Taken together, these findings suggest that [^18^F]MFBG and [^18^F]FDG reflect different aspects of tumor biology, with [^18^F]MFBG showing a particular advantage in depicting the bone marrow disease burden crucial for initial staging.

This study has several limitations. First, it was conducted at a single center included a moderate number of patients. In addition, [^18^F]FDG PET was performed only in a subset, which may have introduced selection bias. Multicenter studies are needed to validate these findings and improve imaging algorithms. Second, [^123^I]MIBG scintigraphy was not included as a directly comparator. Although previous reports have reported higher lesion detection with [^18^F]MFBG compared to [^123^I]MIBG, supporting hNET-targeted PET as a practical functional imaging approach for neuroblastoma 17-20, 32, the present study cannot determine whether the additional findings, stage reclassification, or treatment modifications observed relative to comprehensive anatomic imaging would also have been achieved with standard MIBG-based functional imaging. A prospective head-to-head comparison in newly diagnosed patients is therefore required. Third, the follow-up duration was relatively short (median, 8 months), and further analyses correlating baseline PET findings with event-free survival and outcome measures are necessary.

In summary, hNET-targeted [^18^F]MFBG PET imaging offered valuable additional information during the initial assessment of newly diagnosed neuroblastoma, particularly by enhancing the detection of bone marrow involvement, which impacted staging and treatment decisions in some cases. While lesion-level differences were observed compared with [^18^F]FDG PET, these did not translate into stage discordance. [^18^F] FDG PET may remain valuable in selected clinical scenarios, including suspected hNET-non-avid disease or discordant clinical–imaging findings. These findings demonstrate the additional information provided by hNET-targeted [^18^F]MFBG PET as the baseline functional imaging beyond comprehensive anatomic imaging in newly diagnosed neuroblastoma.

## Figures and Tables

**Table 1:** Demographic and baseline clinical characteristics of patients.

Characteristic	Value, n (%)
**Age, months, median (IQR)**	23.5 (8-43)
**Sex**	
Female	18 (45.0%)
Male	22 (55.0%)
**Serum NSE**	
Elevated	40 (100%)
**Primary tumor site**	
Mediastinum	3 (7.5%)
Retroperitoneum	35 (87.5%)
Pelvis	1 (2.5%)
Not identifiable	1 (2.5%)
**Bone marrow biopsy**	
Positive	11 (27.5%)
Negative	29 (72.5%)
**Genetics**	
MYCN amplification	7 (17.5%)
11q deletion	9 (22.5%)
**Imaging modality**	
PET/CT	28 (70.0%)
PET/MR	12 (30.0%)
**Pathology**	
Ganglioneuroblastoma	5 (12.5%)
Neuroblastoma	35 (87.5%)

**Table 2:** Per-patient lesion detection by anatomic site: anatomic imaging vs [^18^F]MFBG PET.

Anatomic site	Anatomic imaging, n (%)	[^18^F]MFBG PET, n (%)
**Primary tumor**	39 (97.5)	36^ a^ (97.3)
**Regional LN**	32 (80.0)	29 (72.5)
**Distant LN**	15 (37.5)	14 (35.0)
**Bone**	13 (32.5)	26 (65)
Skull	NA^b^	12 (30.0)
Vertebrae	10 (25.0)	19 (47.5)
Pelvis	6 (15.0)	14 (35.0)
Thoracic cage	2 (5.0)	13 (32.5)
Extremities	3 (7.5)	21 (52.5)
**Other sites**	6 (15.0)	6 (15.0)
Liver	4 (10.0)	4 (10.0)
Lung	1 (2.5)	0
kidney	1 (2.5)	1 (2.5)
Pleural	1 (2.5)	1 (2.5)
Skin	0	2 (5.0)

^a^ Three patients underwent resection of the primary tumor before PET; ^b^ NA, not applicable (brain and extremities were outside the anatomic imaging field of view).

## Data Availability

Data generated or analyzed during the study are available from the corresponding author by request.

## Abbreviations

[^18^F]MFBG: [^18^F]meta-fluorobenzylguanidine
[^123^I]MIBG: [^123^I]metaiodobenzylguanidine
[^18^F]FDG: [^18^F]fluorodeoxyglucose
hNET: human norepinephrine transporter
NB: Neuroblastoma
MIP: maximum intensity projection
PET/CT: positron emission tomography-computed tomography
PET/MR: positron emission tomography-magnetic resonance imaging
CAI: comprehensive anatomic imaging
INRG: International Neuroblastoma Risk Group
MIP: Maximum Intensity Projection
